# Bromide Ion Impurity-Induced Reaction between Selenium(IV) and Acidic Bromate: Prototype of a Cycle with Autocatalytic Behavior

**DOI:** 10.1021/acs.inorgchem.3c03833

**Published:** 2024-01-16

**Authors:** György Csekő, Attila K. Horváth

**Affiliations:** Department of General and Inorganic Chemistry, Faculty of Sciences, University of Pécs, Ifjúság útja 6, H-7624 Pécs, Hungary

## Abstract

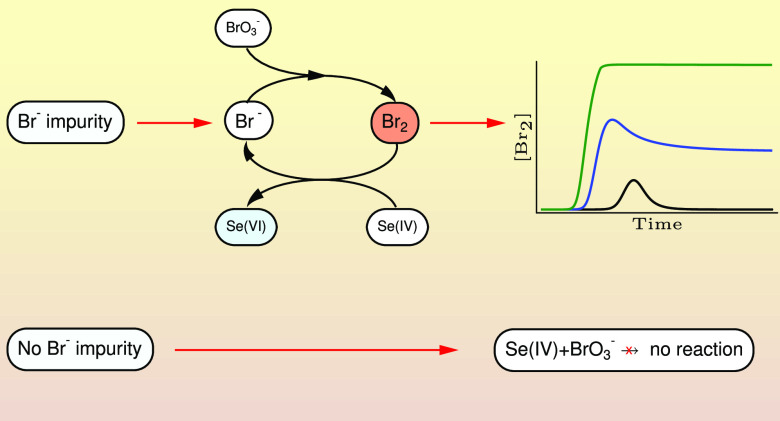

The selenium(IV)–bromate reaction in an acidic medium using phosphoric acid/phosphate buffer was investigated by UV–vis spectroscopy monitoring the formation of bromine. In an excess of bromate, the absorbance–time curves measured at 450 nm display a characteristic sigmoidal shape having a fairly long induction period, while in the opposite case, when selenium(IV) species is used in excess, the measured data follow the rise and fall behavior. Depending on the excess of Se(IV) the final bromine-containing product is either an elementary bromine or bromide ion. Simultaneous evaluation of the measured kinetic traces clearly indicated that, surprisingly, no direct reaction takes place between the reactants. Instead of that, a trace amount of bromide ion impurity in the stock bromate solution is sufficient to drive the system via the oxidation of the bromide ion by bromate producing elementary bromine followed by the subsequent selenite–bromine reaction reestablishing the bromide ion to open a new cycle. As a result, the concentration of bromide ions increases in a sigmoidal fashion during the course of the reaction unless enough selenium(IV) species is present; hence, the overall synergetic effect observed is the autocatalytic rise of bromide ions. Therefore, the cycle mentioned above may be considered as a prototype of autocatalytic cycles. This observation prompted us to clarify the explicit difference between an autocatalytic cycle and an autocatalytic reaction.

## Abstract 2

The apparent selenite−bromate reaction in an acidic medium can be quantitatively interpreted by the unavoidable trace amount of bromide ion impurity present in the stock bromate solution without having any direct reaction between the reactants. The overall bromide−bromate and selenite−bromine systems constitute a cycle with autocatalytic behavior, where the bromide ion acts as a “catalyst” whose concentration increases by the end of each cycle unless enough reducing agent is present.

## Introduction

Selenium is an essential but an ultramicroelement of living organism^1^ and exists in various organic and inorganic compounds.^2^ Main biological effects of this element are dominantly attributed to selenoenzymes containing selenocysteine in their active sites.^3,4^ Even though the necessity of selenium species in nature is inevitable, above a critical level, selenium species are quite toxic.^5^ One of the most harmful inorganic compounds of selenium is the selenite ion and its protonated forms in aqueous solution, although at a low level, moderate selenite treatment of soils positively affected the efficiency of photosynthesis^6^ and increased the survival of rice plants against cadmium pollution.^7^ In addition to that, selenium(IV) may as well behave as an antioxidant^8^ and a prooxidant;^9^ thus, this duality might also play an important role not only in the prevention but also in cancer treatment procedures.^10^ To understand the chemistry behind this versatile behavior of selenium species, it therefore seems to be of special importance to unravel the mechanistic details of their reactions in aqueous conditions. Survey of the literature has, however, revealed only limited examples in studying the kinetics of systems where selenites are involved, in sufficient details. Recently, Dereven’kov and his co-workers investigated the first initiative step leading to the equilibrium formation of thiol-*S*-selenites in the reactions of aliphatic, aromatic and protein thiols with selenite, from which some reactivity–structure relationship was deduced.^11^ This research group also reported a valuable contribution to first step of the glutathione–selenite reaction.^12^ Kinetic and mechanistic patterns of the oxidation of selenium(IV) has been reported by Dikshitulu and Babu.^13^ In addition to that, Liu et al. investigated the kinetic and mechanistic aspects of selenite oxidations by various oxidizing agents like chlorine, bromine, monochloramine, ozone, permanganate, and hydrogen peroxide.^14^ Their major conclusion about the selenite–bromine system seemed to be quite dubious, especially at lower pHs; therefore, Csekő et al. very recently reported the modified kinetic model in the given system that explains quantitatively the pH dependence over a wide pH range of 1–13.^15^ Importance of the latter study is not limited to elucidating the kinetics and mechanism of the selenite–bromine reaction in itself because our preliminary experiments on the selenite–bromate acidic system have revealed the transient formation of bromine in the excess of selenite. As a result, the modified kinetic model must be a crucial part of the mechanism of the title reaction itself. Therefore, here, we report a thorough kinetic investigation of the selenite–bromate system.

## Experimental Section

### Chemicals

All the reagents, including sodium selenite, sodium perchlorate, sodium dihydrogen phosphate, phosphoric acid, sodium bromide, and sodium bromate were analytical grade reagents and used without further purification. No uncommon hazards are noted. The stock solutions were prepared from twice ion-exchanged distilled water which was further distilled twice atmospherically to remove any residues originating from the exchange resin and was also deoxygenated by bubbling through argon gas for at least 20 min. The buffer solutions were made from phosphoric acid, and its exact concentration was determined by classical titrimetry. The pH of the buffer solution was adjusted by the addition of the calculated amount of sodium dihydrogen phosphate by taking the p*K*_a1_ of phosphoric acid to be 1.84.^16^ The selenite, bromate, and bromide stock solutions were prepared by weighing the calculated amount of solid materials. The ionic strength of each solution was adjusted to 0.5 M by adding the necessary amount of sodium perchlorate. It is also crucial to note that only one stock solution was used for the reagents selenite and bromate throughout the whole series of experiments. Later, we will see the importance of this scenario.

### Instrumentation

The kinetic measurements were performed in a standard quartz cuvette equipped with a Teflon cap having an optical path of 1 cm. The Teflon cap was also sealed with Parafilm to minimize the escape of bromine. To maintain the solution homogeneously, magnetic stirrer bars were used (having a length of 8 mm, and a diameter of 2 mm) in each cuvette. The reaction was monitored by a Zeiss S600 diode array spectrophotometer at the wavelength range of 400–900 nm, and every kinetic run contained at least 1000 absorbance–time data pairs originally. All the solutions kept in the cell holder of the instrument were thermostated at 25.0 ± 0.1 °C.

### Kinetic Experiments

Altogether 101 kinetic experiments were performed at different initial concentrations. The concentration ranges used in these experiments were as follows: 3.0–36.6 mM, 3.0–21.0 mM, 0–14.0 μM, and 0.85–1.70 in the cases of selenite, bromate, bromide ion, and the pH, respectively. The solutions were delivered into the cuvette by the following order: first, the buffer solutions were introduced from an Eppendorf pipet followed by the selenite solution. If the Br^–^ dependence of the kinetic curves was studied, then bromide solution was also introduced prior to initiating the kinetic run. Finally, the reaction was initiated by adding the bromate solution from a fast delivery pipet. All the measurements were followed up to at least 95% stoichiometric conversion to gain complete information about the whole course of the reaction.

### Data Treatment and Evaluation

To avoid time-consuming calculations, the number of the absorbance–time data pairs in each kinetic curves has been reduced approximately to 60 based on the principle of equivalent arc length, the method which was described in detail elsewhere.^17^ The absorbance–time traces (measured at 450 nm where only Br_2_ and Br_3_^–^ absorb the light, where ε_Br_2__ =  = 104 M^–1^ cm^–1^^18^) obtained by this way were evaluated by the ChemMech program package developed for determination of the kinetic parameters by minimizing the average absolute deviation between the measured and calculated data-pairs of all the measurements taken into account simultaneously.^19^ The criterion of the best fit was to obtain not more than 2% relative average deviation, which is the experimentally achievable and reasonable limit of error in this system.

## Results

### Characteristics of the Measured Kinetic Curves

**Figure 1:**
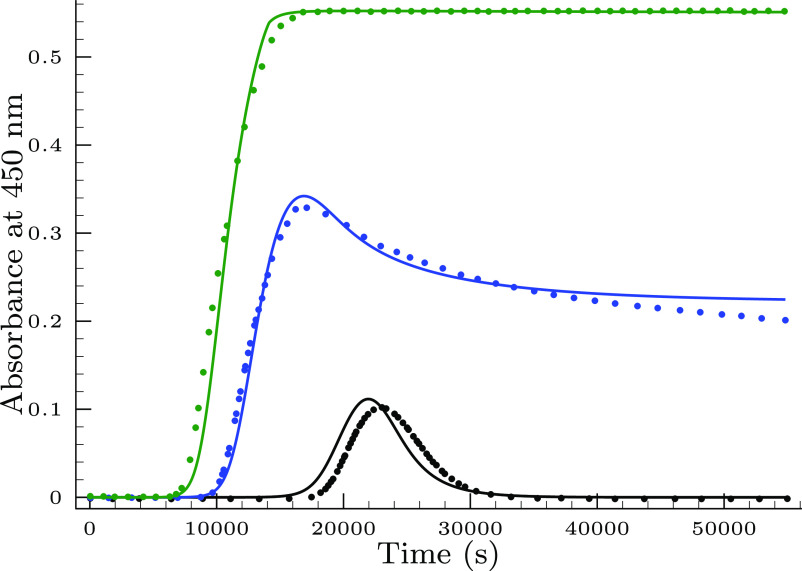
Typical absorbance–time traces measured (color filled circles) in the selenite–bromate reaction. Conditions are as follows: [Se(IV)]_*T*,0_ = 26.8 mM, pH = 1.1, [BrO_3_^–^]_0_/mM = 5.7 (black), 9.7 (blue), and 12.8 (green). The solid lines represent the fitted absorbance–time curves by the proposed model shown in Table 1.

Figure 1 displays three types of typical kinetic traces. All of them starts with a fairly long, but reproducible induction period followed by a relatively rapid rise of the absorbance. Depending on the initial concentration ratio of the reactants, the absorbance–time curve either levels off or the absorbance start to decrease. Extent of the declining phase also depends on the total selenite concentration. When [Se(IV)]_*T*,0_ is high enough, then the absorbance decreases to zero; otherwise, it may level off at a significantly lower value compared to the absorbance maximum.

**Figure 2:**
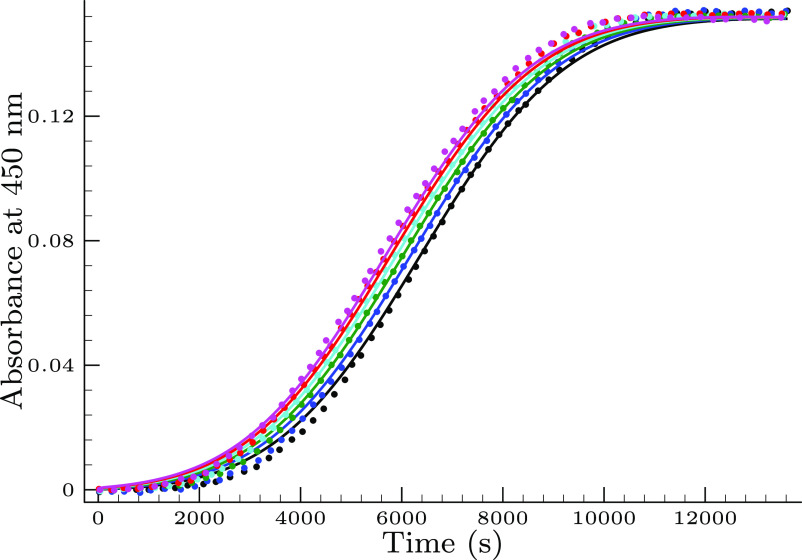
Effect of the initially added bromide ion on the kinetic traces in the selenite–bromate reaction. Conditions are as follows: [Se(IV)]_*T*,0_ = 7.3 mM, pH = 0.95, [BrO_3_^–^]_0_ = 15.0 mM, and [Br^–^]_0_/μM = 4.0 (black), 6.0 (blue), 8.0 (green), 10.0 (cyan), 12.0 (red), and 14.0 (magenta). The solid lines represent the fitted absorbance–times curves by the proposed model.

This behavior remarkably resembles to the fingerprint of autocatalysis-driven clock reactions.^20^ Indeed, as it was shown recently, the selenite–bromine reaction is a relatively slow process around pH = 1.0 due to H^+^-inhibition^15^ and therefore it becomes commensurable with the relatively sluggish bromide–bromate reaction especially at lower pHs.^21−26^ If our assumption regarding the classification of this system to be designated as an autocatalysis-driven clock reaction is correct, then we may expect that the bromide ion acts as an autocatalyst in the title system. Figure 2 displays the effect of the initially added bromide ion on the absorbance–time profiles.

It clearly proves that the bromide ion shortens the induction period and facilitates the formation of bromine, indicating an autocatalytic nature of the title system.^27^

### Stoichiometry

The measurements presented here strongly suggest that the stoichiometry of title reaction is regulated by the following limiting stoichiometries [note that in eqs 1 and 2 SeO_3_^2–^ corresponds to all selenium(IV) species irrespective to its actual speciation]
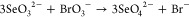
1and
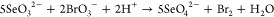
2

Consequently, it means that huge excess of bromate prefers eq 2, hence, the formation of bromine, while selenite excess will gradually remove bromine to reestablish the bromide ion leading to the limiting stoichiometry represented by eq 1. It means that the actual stoichiometry of the title reaction varies with the initial concentration of the reagents and can be described by the linear combination of eqs 1 and 2 under the experimental conditions applied here.

### Construction of the Proposed Kinetic Model

Our first trial for simultaneous description of the kinetic curves consisted of three overall steps similar to those used in the case of Landolt-type reactions.^20^ The reaction is supposed to start with the successive possibly oxygen-transfer processes from bromate to selenite eventually to yield bromide and selenate ions (see: eq 1). Then the bromide ion is oxidized by bromate to produce elementary bromine
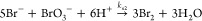
3

To complete the cycle, selenite reduces bromine back to bromide ion via the following step
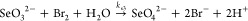
4

The kinetics of eq 4 was found to be very complex, recently an overall model was proposed to describe all the characteristic features of this reaction including hydrogen and bromide inhibited pathways.^15^ It was also demonstrated that this mechanism is capable of explaining the pH-dependence of the reaction at the pH range of 1–13. Therefore, here, we directly implemented it in the proposed model without any simplification to preserve all the characteristics of the selenite–bromine reaction. This part of the proposed model is represented in Table S1 of the Supporting Information. As a next step, we considered eq 3 with the most widely accepted rate law^21−25^
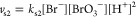
5

Finally, the direct reaction between the reactants was considered as
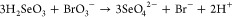
6with the following rate law
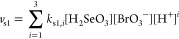
7

Consequently, the kinetic model consisted of all the reactions with their corresponding rate laws found in Table S1, eq 6 with the rate of eqs 7 and 3 with that of eq 5. *k*_s1,*i*_ and *k*_s2_ values were optimized during the whole course of the data evaluation process, but all the rate coefficients presented in Table S1 of the Supporting Information were fixed. As a result, we obtained the best fit with *k*_s1,2_ = 0.019 ± 0.01 M^–3^ s^–1^ and *k*_s2_ = 33.2 ± 0.4 M^–3^ s^–1^, but the average deviation between the measured and calculated data were found to be 7.3% which was unacceptably high, indicating that the kinetic model should be further improved.

Taking into consideration that the initiation step is very slow, as a next step, we omitted eq 6 from the kinetic model and supposed that the stock bromate solution contained trace amount of bromide ion impurity. This is not uncommon at all because in the case of the arsenous acid–iodate reaction, it was a general belief until 2016^28^ that there is no direct reaction between these reagents and, in fact, unavoidable trace amount of iodide ion impurity of the iodate stock solution drives the overall system.^29,30^ One may safely assume on the basis of halogenate analogy that in the case of bromate stock solution, the bromide ion is also an unavoidable impurity present in a very small amount. Our experimental setup along with the ChemMech program package made it possible to execute simultaneous evaluation of the kinetic curves by adjusting just the bromide ion impurity of the stock bromate solution from which for each experimental run, the actual initial bromide ion concentration can be calculated automatically by simple dilution. Thus, in the second trial, we fitted [Br^–^]_0_ of the bromate stock solution along with eq 3 incorporated with its rate law shown in eq 5. Note that reactions with their corresponding rate laws and rate coefficients shown in Table S1 are also included. Surprisingly, a better but still not acceptable average deviation is obtained with a relative error of 5.0% by the values of [Br^–^]_0_ = (5.3 ± 3.4) × 10^–19^ M and *k*_s2_ = 120 ± 2 M^–3^ s^–1^. From these outcomes, we concluded that eq 3 in itself with its widely accepted rate equation is insufficient to describe our measured data. Therefore, as a next step, we considered the overall bromide–bromate reaction to be driven via the Br_2_O_2_ short-lived intermediate, which was suggested by Schmitz^31^ from the generalization of the mechanisms of halogenate–halide reactions
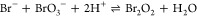
8with the corresponding rate laws for both the forward and the backward directions shown below
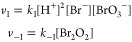
9

This intermediate is then ready to react rapidly with selenium(IV) species to give the products selenate and hypobromous acid as follows
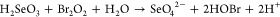
10

Its rate law was supposed to be the following
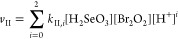
11which provides a straightforward possibility to check the influence of pH on this reaction as well. For the sake of completeness, it is very important to emphasize that hypobromous acid formed in eq 10 is not a final product of the overall reaction because it is removed via the reactions with selenite ion and hydrogen-selenite ion or indirectly via bromine hydrolysis along with the selenium(IV)–bromine system displayed in Table S1. Consequently, it means that *k*_I_ and *k*_II,*i*_ values and the initial bromide concentration of the stock bromate solution were fitted during the course of the data evaluation process, keeping all the rate coefficients found in Table S1 and *k*_–I_ = 100 s^–1^ fixed. The necessity of the latter one to hold it unchanged at this value is to keep the steady-state concentration of Br_2_O_2_ at low concentrations not to accumulate this species at a detectable concentration. As a result, we have obtained again a significantly better average deviation (4.0%) between the measured and calculated data. Even though this value was found to be so far the best one, still comparison of the measured and calculated data revealed systematic differences especially at higher pHs (some representative examples are shown in Figure S1 of the Supporting Information.) It clearly suggested that the kinetic model should further be improved. The best result was obtained when *k*_I_ = 96 ± 1 M^–3^ s^–1^, [Br^–^]_0_ = (5.5 ± 2.3) × 10^–18^ M and basically the quality of the fit did not change if *k*_II,1_ > 6 × 10^11^ M^–2^ s^–1^ inequality is fulfilled; hence, it means that this reaction has to be considered as a rapid process under these conditions.

In our next probe, we have included the reaction of the reactive intermediate Br_2_O_2_ into the previous kinetic model
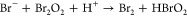
12with the following rate equation
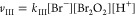
13

This reaction was suggested by Schmitz^26^ in explaining the formal kinetic order of bromide ion to be higher than one in the case of the bromide–bromate reaction. Of course, bromous acid cannot be accumulated as a final product in the title reaction; therefore, we also inserted the following, possibly oxygen-transfer, reaction between selenium(IV), and bromous acid
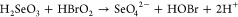
14having the rate law of
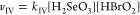
15

**Figure 3:**
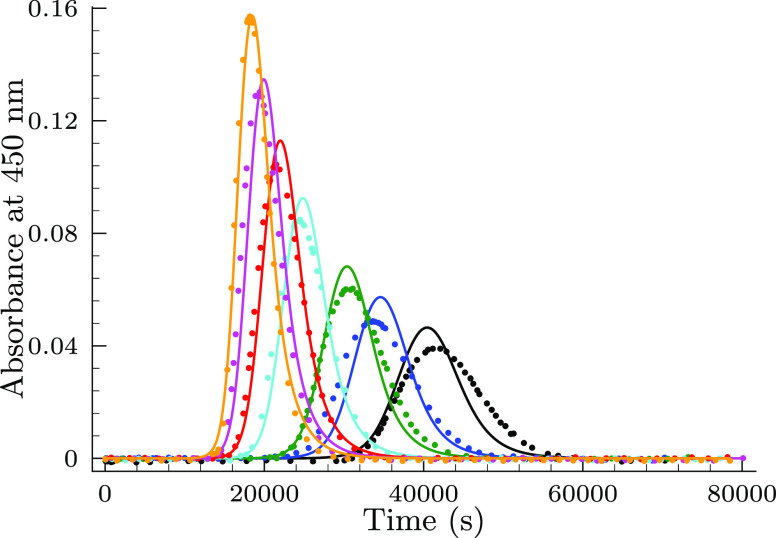
Measured (symbols) and calculated (solid lines) absorbance–time profiles in the selenite–bromate reaction keeping the ratio of [Se(IV)]_*T*,0_/[BrO_3_^–^]_0_ = 14:3 at pH = 1.1 in the absence of the bromide ion. [BrO_3_^–^]_0_ = 3.0 mM (black), 3.34 (blue), 4.2 (green), 5.0 (cyan), 5.73 (red), 6.75 (magenta), and 7.8 (orange). The solid lines represent the fitted absorbance–time curves by the proposed model shown in Table 1.

**Figure 4:**
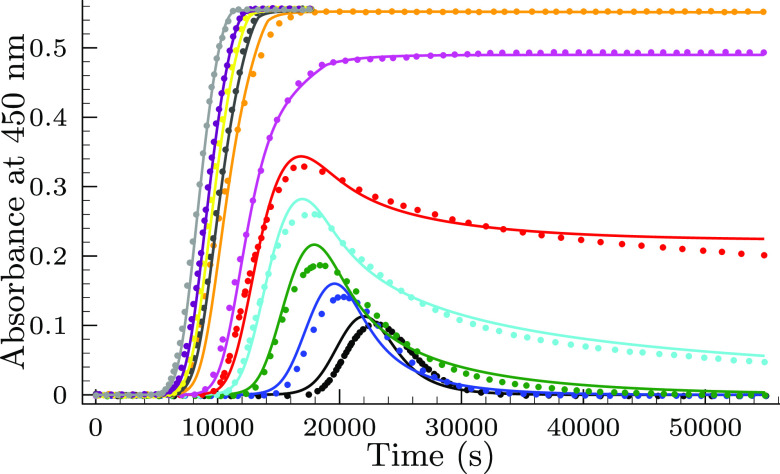
Measured (symbols) and calculated (solid lines) absorbance–time profiles in the selenite–bromate reaction at a constant selenite concentration ([Se(IV)]_*T*,0_ = 26.8 mM) in the absence of initially added bromide ion at pH = 1.1. [BrO_3_^–^]_0_ = 5.7 mM (black), 6.75 (blue), 7.8 (green), 8.9 (cyan), 9.75 (red), 10.5 (magenta), 12.75 (orange), 13.5 (dark gray), 14.25 (yellow), 15.0 (purple), and 16.5 (light gray). The solid lines represent the fitted absorbance–times curves by the proposed model shown in Table 1.

**Figure 5:**
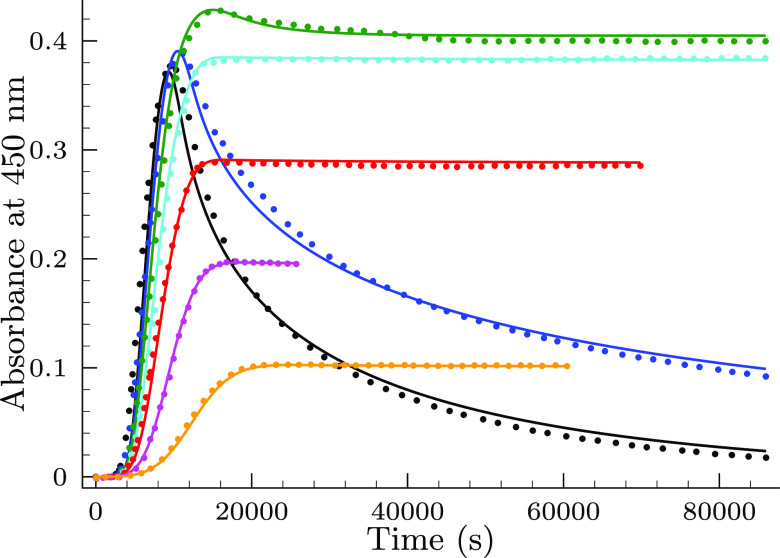
Measured (symbols) and calculated (solid lines) absorbance–time profiles in the selenite–bromate reaction at a constant bromate concentration ([BrO_3_^–^]_0_ = 9.0 mM) in the absence of initially added bromide ion at pH = 0.85. [Se(IV)]_*T*,0_ = 32.2 mM (black), 27.6 (blue), 23.1 (green), 18.6 (cyan), 14.0 (red), 9.5 (magenta), and 5.0 (orange). The solid lines represent the fitted absorbance–times curves by the proposed model shown in Table 1.

**Figure 6:**
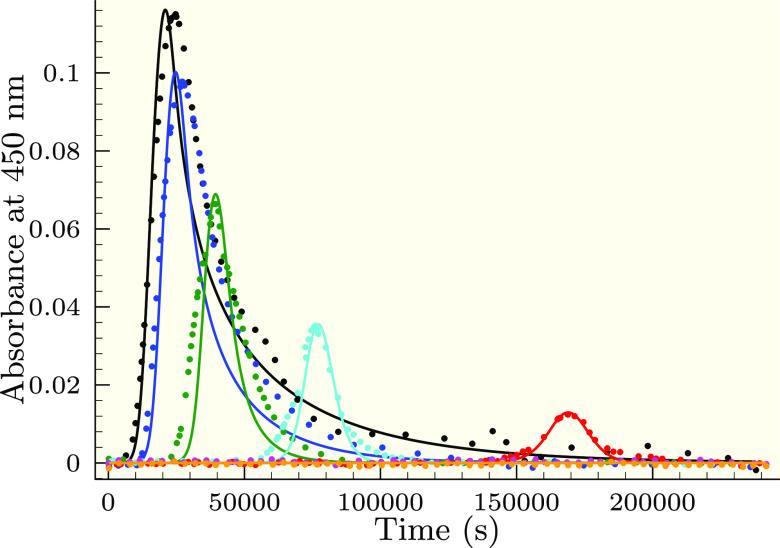
Measured (symbols) and calculated (solid lines) absorbance–time profiles in the selenite–bromate reaction with varying the pH in the absence of the initially added bromide ion. Conditions: [Se(IV)]_*T*,0_ = 10.7 mM and [BrO_3_^–^]_0_ = 3.0 mM. pH = 0.85 (black), 0.95 (blue), 1.1 (green), 1.25 (cyan), 1.4 (red), 1.55 (magenta), and 1.7 (orange). The solid lines represent the fitted absorbance–times curves by the proposed model shown in Table 1.

As a result, in our next trial, *k*_I_, *k*_II,1_, *k*_III_ parameters and the initial bromide concentration of the stock bromate solution were fitted, but *k*_–I_ = 100 s^–1^ and *k*_IV_ = 10^7^ M^–1^ s^–1^ parameters were fixed during the course of the evaluation process. It is also worthwhile to note that the data found in Table S1 were also included. As a surprise, a strikingly good average deviation in terms of relative error is obtained to be 1.3% between the experimental and calculated data with the following fitted parameters: *k*_I_ = 71.5 ± 0.3 M^–3^ s^–1^, *k*_II,1_ = (2.74 ± 0.01) × 10^5^ M^–2^ s^–1^, *k*_III_ = (1.28 ± 0.08) × 10^7^ M^–2^ s^–1^, and [Br^–^]_0_ = (2.55 ± 0.30) × 10^–11^ M. Figures 3–6 clearly indicate that the present model is capable of a sound description of the experimental data measured under various initial conditions.

## Discussion

Table 1 shows the proposed model including all of the necessary information that enables anyone to reproduce the fitted kinetic traces.

**Table 1:** Proposed Kinetic Model^a^

step	reaction	rate law	rate coefficients
(I)	Br^–^ + BrO_3_^–^ + 2H^+^ ⇌ Br_2_O_2_ + H_2_O	*v*_I_ = *k*_I_[Br^–^][BrO_3_^–^][H^+^]^2^	71.5 ± 0.3 M^–3^ s^–1^
		*v*_–I_ = *k*_–I_[Br_2_O_2_]	100 s^–1^
(II)	H_2_SeO_3_ + Br_2_O_2_ + H_2_O → SeO_4_^2–^ + 2HOBr + 2H^+^	*v*_II_ = *k*_II,1_[H_2_SeO_3_][Br_2_O_2_][H^+^]	(2.74 ± 0.01) × 10^5^ M^–2^ s^–1^
(III)	Br^–^ + Br_2_O_2_ + H^+^ → Br_2_ + HBrO_2_	*v*_III_ = *k*_III_[Br^–^][Br_2_O_2_][H^+^]	(1.28 ± 0.08) × 10^7^ M^–2^ s^–1^
(IV)	H_2_SeO_3_ + HBrO_2_ → SeO_4_^2–^ + HOBr + 2H^+^	*v*_IV_ = *k*_IV_[H_2_SeO_3_][HBrO_2_]	>10^7^ M^–1^ s^–1^

a Note that for a complete description, data in Table S1 are necessary. The bromide impurity for the stock 0.048 M BrO_3_^–^ solution was found to be [Br^–^]_0_ = (2.55 ± 0.30) × 10^–11^ M. All the reactions for Se(IV)–Br_2_ system shown in Table S1.

As it is seen the proposed model does not require the presence of direct reaction (see: eq 6) for quantitative description of the measured data. To confirm further this observation we have carried out an additional fitting procedure, where the bromide ion impurity of the stock bromate solution was neglected (see: Table 1) and replaced by eq 6 to initiate the title reaction. The average deviation was found to be 1.4%, indicating that this modified model also provides a sound description of the measured kinetic traces. Moreover, for the *k*_I_, *k*_II,1_, and *k*_III_ values, we obtained 71.9 ± 0.2 M^–3^ s^–1^, (2.87 ± 0.04) × 10^5^ M^–2^ s^–1^, and (1.90 ± 0.05) × 10^7^ M^–2^ s^–1^, respectively, meaning that they essentially remain the same as found in the proposed kinetic model (see Table 1). However, in this case, the calculated value of *k*_s1_ = (7.14 ± 0.13) × 10^–10^ M^–2^ s^–1^ indicates that the direct reaction has to be a vanishingly slow process. In other words, it means that at pH = 1.0 and at large bromate excess (0.1 M) the half-life of selenium(IV) species would approximately be 3080 years! From this, one can safely conclude that practically this reaction does not occur at all. To support this conclusion, one should also take into consideration that the trace amount of bromide impurity of bromate solution cannot be avoided technically; thus, the value of *k*_s1_ has to be clearly overestimated. Therefore, the real question is whether the bromide impurity alone drives the reaction or the unavoidable trace amount of the bromide ion present in the stock bromate solution along with the vanishingly slow direct reaction. According to Occam’s razor among two competing possibilities, always the simpler explanation is to be preferred; hence, it is quite reasonable to assume that the title reaction is initiated by the bromide ion impurity of the stock bromate solution.

Such a sensitive dynamic system also poses an important question about the reproducibility of the kinetic curves. We emphasize again that the whole series of experimental traces was executed from the same bromate stock solution, which means that the bromide contamination of each individual run was vigorously controlled by bromide ion concentration present in the stock solution. Thus, everyone may straightforwardly expect that the kinetic traces are completely reproducible if this is so. Figure S2 of the Supporting Information displaying triplicate experiments clearly confirms sound reproducibility. This finding also poses an important question about the reproducibility of the kinetic curves if they are performed from physically different bromate stock solutions having the same initial concentration of BrO_3_^–^. We expect that it is difficult, almost impossible, to control such a low level of bromide ion impurity for both stock solutions; thus, the kinetic curves may be shifted along the time-axis by a notable level. Indeed, this expectation was confirmed as indicated in Figure S3 of the Supporting Information. All of these important findings strongly support our experimental setup of using the same bromate stock solution via the whole series of kinetic runs, which enabled us to evaluate the measured data simultaneously and at the same time determine the bromide impurity of the stock bromate solution used, unambiguously. Otherwise, it would have led to a much more complicated situation to exploit the advantages of simultaneous data evaluation.

It is also important to note that the bromide impurity of the bromate stock solution determined from simultaneous evaluation of the kinetic curves is orders of magnitude lower than the deliberately used bromide concentration to confirm the autocatalytic nature of the reaction (see: Figure 2). Thus, one would easily expect that the induction period would be diminished upon applying such a relatively huge concentration of bromide ion compared to its impurity level. As it is seen in Figure 2 it is not the case, the induction period just slightly decreases upon using a micromolar amount of bromide ion. This apparent contradiction between the expectation and the real kinetic behavior may easily be reconciled if one takes into consideration that in the complete absence of bromide ion no bromine formation occurs at all; consequently, the “induction period” would be infinitely long. In other words, it means that using micromolar initial bromide concentration indeed significantly decreases the length of the induction period compared to the case when no bromide ion is present at all.

Finally, a paragraph is also in an order here to clarify why it is better to use the term autocatalytic cycle^32^ rather than autocatalytic reaction for the selenite–bromate system. First, in an ideal case, where bromide ion free bromate and selenium(IV) species are mixed, no reaction would take place at all. However, trace amounts of bromide ion are always present in an aqueous bromate solution; thus, one will observe the oxidation of selenite to selenate. This process, however, occurs via the sequence of bromide–bromate and selenium(IV)–bromine reactions forming a catalytic cycle with respect to the bromide ion opening up an alternative route for the title reaction instead of the direct oxidation which was proven to be nonexisted or at least vanishingly slow. As a result, by the end of this cycle the bromide ion continuously increases meaning that bromide ion will accelerate its own formation resulting in the appearance of autocatalytic behavior. In other words the reaction, between the otherwise unreactive selenium(IV) and bromine(V) species toward each other, is driven by the assistance of bromine formation and disappearance from eqs 3 and 4 resulting eventually in an autocatalytic cycle. If one insists on using the term “autocatalytic reaction”, we suggest that the “autocatalytic selenite–bromate–bromide reaction” instead of the “autocatalytic selenite–bromate reaction” would thus be a better choice.

## Conclusions

In this article, it is clearly demonstrated that even though bromine, depending on the excess of reducing or oxidizing agents, may either appear as a transient species or a final product, respectively, after a well-defined and reproducible time lag during the course of the reaction. This experimental finding may be unintentionally but falsely misidentified as the given system resembles to Landolt-type reactions.^20^ Instead of that the reaction is in fact initiated by the unavoidable trace amount of bromide ion impurity originating from the bromate stock solution. This tiny or almost negligible amount is enough to maintain a cycle in which elementary bromine formed via the bromide–bromate reaction oxidizes selenite to selenate ion; meanwhile, it is reduced back to the bromide ion to start a new cycle for continuous removal of the reactants unless one of them is completely depleted closing a cycle. Increasing initial bromide concentration significantly decreases the length of induction period paving the way to identify the above-mentioned cycle as an “autocatalytic cycle”.^32^ Even though autocatalytic systems where photoinduction is responsible for the initiation were already reported in the case of two-component reactions,^33,34^ to the best of our knowledge this is the first real chemical system, where two reactants are consumed in an apparent chemical reaction in an autocatalytic fashion by the assistance of a third species without having any direct interaction between the reactants.

## Supporting Information Available

Complete kinetic model of the selenium(IV)–bromine reaction, results of poor modeling by neglecting the Br–Br_2_O_2_ reaction, and reproducibility and time shift of the kinetic curves when initiating from the same and different stock bromate solutions (PDF)
