# Supplementary material for: Bromide Ion Impurity-Induced Reaction between Selenium(IV) and Acidic Bromate: Prototype of a Cycle with Autocatalytic Behavior
Source: Inorg Chem. 2024 Jan 16;63(4):2053–9. doi: 10.1021/acs.inorgchem.3c03833 (PMC10828994; doi:10.1021/acs.inorgchem.3c03833)
Supplement: Supplementary file 1 — ic3c03833_si_001.pdf [file ic3c03833_si_001.pdf]

Supporting Information for Bromide Ion Impurity Induced Reaction Between Selenium(IV) and Acidic Bromate: Prototype of a Cycle With Autocatalytic Behavior

György Csekő and Attila K. Horváth**

Department of General and Inorganic Chemistry, Faculty of Sciences, University of Pécs, Ifjúság útja 6., H-7624 Pécs, Hungary

E-mail: horvatha@gamma.ttk.pte.hu

Table S1: Complete kinetic model for the selenite–bromine reaction. The discussion about the rate laws presented here may be found in reference 15 (Csekő et al., ACS Omega, 2023, 8, 15769.)

Step	Reaction	Rate Laws	Rate coefficients
(1)	$SeO_3^{2-} + H^+ \rightleftharpoons HSeO_3{}^-$	$k_1[SeO_3^{2-}][H^+]$	10^{10} $M^{-1}s^{-1}$
		$k_{-1}[HSeO_3^-]$	138 s^{-1}
(2)	$HSeO_3^- + H^+ \rightleftharpoons H_2SeO_3$	$k_2[HSeO_3^-][H^+]$	10^{10} $M^{-1}s^{-1}$
		$k_{-2}[H_2SeO_3]$	4.9×10^7 s^{-1}
(3)	$SeO_3^{2-} + HSeO_3^- \rightleftharpoons H(SeO_3)_2^{3-}$	$k_3[SeO_3^{2-}][HSeO_3^-]$	10^{10} $M^{-1}s^{-1}$
		$k_{-3}[H(SeO_3)_2^{3-}]$	3.89×10^9 s^{-1}
(4)	$SeO_3^{2-} + H_2SeO_3 \rightleftharpoons H_2(SeO_3)_2^{2-}$	$k_4[SeO_3^{2-}][H_2SeO_3]$	10^{10} $M^{-1}s^{-1}$
		$k_{-4}[H_2(SeO_3)_2^{2-}]$	1.1×10^4 s^{-1}
(5)	$H^+ + H_2(SeO_3)_2^{2-} \rightleftharpoons H_3(SeO_3)_2^-$	$k_5[H^+][H_2(SeO_3)_2^{2-}]$	10^{10} $M^{-1}s^{-1}$
		$k_{-5}[H_3(SeO_3)_2^-]$	1.32×10^7 s^{-1}
(6)	$H^+ + H_3(SeO_3)_2^- \rightleftharpoons H_4(SeO_3)_2$	$k_6[H^+][H_3(SeO_3)_2^-]$	10^{10} $M^{-1}s^{-1}$
		$k_{-6}[H_4(SeO_3)_2]$	5.75×10^7 s^{-1}
(7)	$Br_2 + H_2O \rightleftharpoons HOBr + H^+ + Br^-$	$k_7[Br_2]$	97 s^{-1}
		$k_{-7}[HOBr][H^+][Br^-]$	1.6×10^{10} $M^{-2}s^{-1}$
(8)	$Br_2 + H_2PO_4^- + H_2O \rightleftharpoons HOBr + Br^- + H_3PO_4$	$k_8[Br_2][H_2PO_4^-]$	10^3 $M^{-1}s^{-1}$
		$k_{-8}[HOBr][Br^-][H_3PO_4]$	2.4×10^9 $M^{-2}s^{-1}$
(9)	$Br_2 + Br^- \rightleftharpoons Br_3^-$	$k_9[Br_2][Br^-]$	1.81×10^9 $M^{-1}s^{-1}$
		$k_{-9}[Br_3^-]$	10^8 s^{-1}
(10)	$H_3PO_4 \rightleftharpoons H^+ + H_2PO_4^-$	$k_{10}[H_3PO_4]$	1.45×10^8 s^{-1}
		$k_{-10}[H^+][H_2PO_4^-]$	10^{10} $M^{-1}s^{-1}$
(I)	$HSeO_3^- + Br_2 \rightleftharpoons SeO_3Br^- + Br^- + H^+$	$k_I[HSeO_3^-][Br_2]$	8.32 $M^{-1}s^{-1}$
		$k_{-I}[SeO_3Br^-][Br^-][H^+]$	10^8 $M^{-2}s^{-1}$
(II)	$SeO_3Br^- + H_2O \rightarrow SeO_4{}^{2-} + Br^- + 2H^+$	$k_{II}[SeO_3Br^-][H^+]^{-1}$	257 Ms^{-1}
		$k_{-II}[SeO_3Br^-][Br^-][H^+]^{-4}$	0.0895 M^3s^{-1}
(III)	$HSeO_3{}^- + HOBr \rightarrow SeO_4{}^{2-} + Br^- + 2H^+$	$k_{III}[HSeO_3{}^-][HOBr]$	700 $M^{-1}s^{-1}$
(IV)	$SeO_3{}^{2-} + HOBr \rightarrow SeO_4{}^{2-} + Br^- + H^+$	$k_{IV}[SeO_3{}^{2-}][HOBr]$	44000 $M^{-1}s^{-1}$

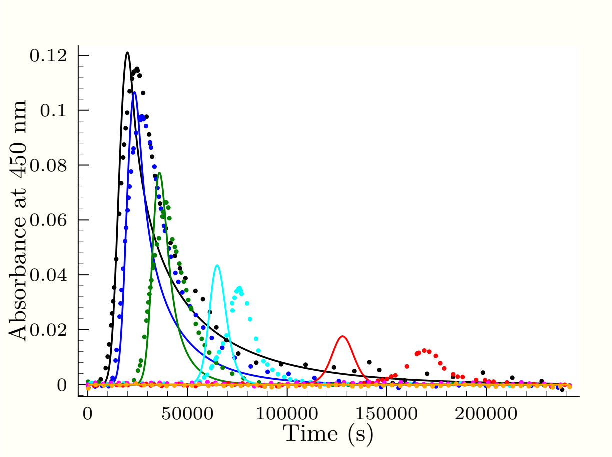

Figure S1: Measured (symbols) and calculated (solid lines) absorbance–time profiles in the selenite–bromate reaction with varying the pH in the absence of initially added bromide ion. Conditions: $[\mathrm{Se(IV)}]_{T,0} = 10.7$ mM and $[\mathrm{BrO_3}^-]_0 = 3.0$ mM. pH = 0.85 (black), 0.95 (blue), 1.1 (green), 1.25 (cyan), 1.4 (red), 1.55 (magenta) and 1.7 (orange). The calculation was performed when bromide impurity of the bromate stock solution was taken into consideration. The kinetic model consisted of Table S1 along with eqs. 8 and 10 of the manuscript with their corresponding rate equations (eqs. 9 and 11). The low quality of the fit justifies that different kinetic model should be used.

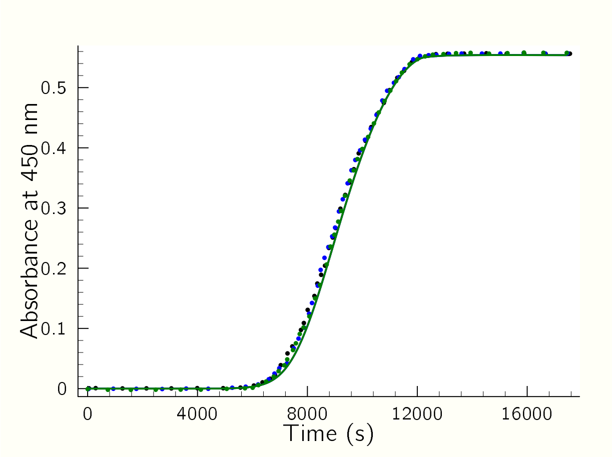

Figure S2: Triplicate experiments in the selenite–bromate reaction indicating a perfect reproducibility when the reaction was initiated from the same bromate stock solution. Conditions: $[\mathrm{Se(IV)}]_{T,0} = 26.8$ mM and $[\mathrm{BrO_3}^-]_0 = 15.0$ mM and pH = 1.1. Experiment 1 (black), Experiment 2 (blue) and Experiment 3 (green).

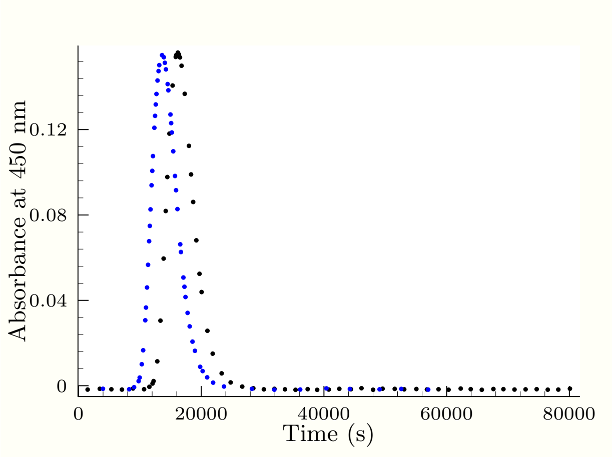

Figure S3: Duplicate experiments in the selenite–bromate reaction, when the kinetic runs were initiated from physically different bromate stock solutions having the same concentration indicating different initial bromide concentration impurity of stock solutions. Conditions: $[\mathrm{Se(IV)}]_{T,0} = 36.6$ mM and $[\mathrm{BrO_3}^-]_0 = 7.8$ mM and pH = 1.1. Experiment 1 (black) and Experiment 2 (blue). Estimated bromide impurity from the kinetic model presented is: 9.3×10^{-11} (black) and 3.5×10^{-9} M (blue).
